# Effects of late, repetitive remote ischaemic conditioning on myocardial strain in patients with acute myocardial infarction

**DOI:** 10.1007/s00395-022-00926-7

**Published:** 2022-04-23

**Authors:** J. Ranjit Arnold, Andrew P.Vanezis, Glenn C. Rodrigo, Florence Y. Lai, Prathap Kanagala, Sheraz Nazir, Jamal N. Khan, Leong Ng, Kamal Chitkara, J. Gerry Coghlan, Simon Hetherington, Nilesh J. Samani, Gerald P. McCann

**Affiliations:** 1 Department of Cardiovascular Sciences, University of Leicester, National Institute for Health Research (NIHR) Leicester Biomedical Research Centre, Glenfield Hospital, Groby Road, Leicester, LE3 9QP UK; 2 Liverpool University Hospitals NHS Foundation Trust, Liverpool, UK; 3 Royal Derby Hospital, Derby, UK; 4 Royal Free Hospital, London, UK; 5 Kettering General Hospital, Kettering, UK

**Keywords:** Heart failure, Primary percutaneous coronary intervention, Remodelling, Remote ischaemic conditioning, ST elevation myocardial infarction, Strain

## Abstract

Late, repetitive or chronic remote ischaemic conditioning (CRIC) is a potential cardioprotective strategy against adverse remodelling following ST-segment elevation myocardial infarction (STEMI). In the randomised Daily Remote Ischaemic Conditioning Following Acute Myocardial Infarction (DREAM) trial, CRIC following primary percutaneous coronary intervention (P-PCI) did not improve global left ventricular (LV) systolic function. A post-hoc analysis was performed to determine whether CRIC improved regional strain. All 73 patients completing the original trial were studied (38 receiving 4 weeks’ daily CRIC, 35 controls receiving sham conditioning). Patients underwent cardiovascular magnetic resonance at baseline (5–7 days post-STEMI) and after 4 months, with assessment of LV systolic function, infarct size and strain (longitudinal/circumferential, in infarct-related and remote territories). At both timepoints, there were no significant between-group differences in global indices (LV ejection fraction, infarct size, longitudinal/circumferential strain). However, regional analysis revealed a significant improvement in longitudinal strain in the infarcted segments of the CRIC group (from − 16.2 ± 5.2 at baseline to − 18.7 ± 6.3 at follow up, p = 0.0006) but not in corresponding segments of the control group (from − 15.5 ± 4.0 to − 15.2 ± 4.7, p = 0.81; for change: − 2.5 ± 3.6 versus + 0.3 ± 5.6, respectively, *p* = 0.027). In remote territories, there was a lower increment in subendocardial circumferential strain in the CRIC group than in controls (− 1.2 ± 4.4 versus − 2.5 ± 4.0, *p* = 0.038). In summary, CRIC following P-PCI for STEMI is associated with improved longitudinal strain in infarct-related segments, and an attenuated increase in circumferential strain in remote segments. Further work is needed to establish whether these changes may translate into a reduced incidence of adverse remodelling and clinical events. Clinical Trial Registration: http://clinicaltrials.gov/show/NCT01664611.

## Introduction

The decline in mortality following ST-segment elevation myocardial infarction (STEMI) is mirrored by an increasing number of survivors with residual heart failure, a condition whose prognosis has not improved significantly in the last two decades [43, 57]. Therefore, key goals in combating ischaemic heart failure include (1) the early identification of at-risk individuals and (2) the development of effective strategies to limit adverse left ventricular (LV) remodelling.

Remote ischaemic conditioning (RIC) is a non-invasive cardioprotective strategy delivered through serial, short-lived periods of ischaemia–reperfusion in a tissue bed remote from the heart [19, 22, 40]. In some previous studies undertaken during the acute phase of STEMI, adjunctive RIC (with primary percutaneous coronary intervention [P-PCI]) attenuated infarct size and/or reduced the incidence of LV systolic dysfunction and major adverse cardiac events, albeit not consistently [1, 5, 9, 14, 31, 32, 42, 56, 60]. However, in the largest-scale randomised trial (CONDI-2/ERIC-PPCI, *n* = 5401), RIC failed to reduce infarct size or improve 12 month clinical outcomes (cardiac mortality/heart failure hospitalisation) [13, 18].

It has been suggested that a multi-targeted approach combining RIC with postconditioning immediately after stenting may afford greater cardioprotection [6, 8, 50]. Animal studies have also shown that late, repetitive ‘chronic’ remote ischaemic conditioning (CRIC) may mitigate against adverse LV remodelling [55]. We carried out the first randomised clinical trial evaluating CRIC (commencing on the third day following successful P-PCI and administered daily for 4 weeks) [51]. However, cardiovascular magnetic resonance (CMR) demonstrated no effect of CRIC on infarct size or global volumetric indices. Although CRIC commenced late after infarction may not be expected to impact infarct size, it may influence cardiac remodelling: hence, in this post-hoc analysis, we evaluated regional cardiac function as determined by myocardial strain imaging.

## Methods

Our study was based on a non-specified post-hoc analysis of 73 STEMI patients recruited in the multicentre, prospective randomised Daily Remote Ischaemic Conditioning Following Acute Myocardial Infarction (DREAM) trial, assessing the impact of CRIC on LV systolic function. Trial design and methodology are as previously reported [51]. All subjects studied in the main trial were included in this post-hoc analysis. In brief, the trial comprised patients presenting to 4 P-PCI centres with a first STEMI successfully treated with P-PCI and baseline LVEF < 45% (determined by echocardiography). Participants were randomised in a 1:1 ratio (stratified by age, gender and infarct location) to 4 weeks’ duration of CRIC or sham treatment, beginning on the third day post-MI and self-administered at participants’ homes using the autoRIC^®^ Device (CellAegis Devices Inc, Toronto, Canada). The device was applied to the upper arm and for CRIC, the device delivered four 5 min cycles of inflation at 200 mmHg separated by 5 min of deflation between each cycle [total treatment time 35 min]; in the control group, the sham device employed the same cycle durations with inflation to 10 mmHg. In both groups during inflation, the device made identical vibrating noises, and participants were not told of the level of inflation required to deliver active treatment and whether they were in the control group. Participants were instructed to apply the device at the same time of day and on the same arm. Participants were asked to keep a diary of use of the device and were removed from the trial if they returned an incomplete diary sheet, missed more than three treatment sessions or missed more than two consecutive days of treatments. Written informed consent was obtained from each patient prior to enrolment. The study was approved by the regional ethics committee (12/EM/0304) and registered with clinicaltrials.gov (NCT01664611). It was conducted according to the principles of Good Clinical Practice and according to the Declaration of Helsinki, under the oversight of the University of Leicester.

### CMR imaging

**Fig. 1:**
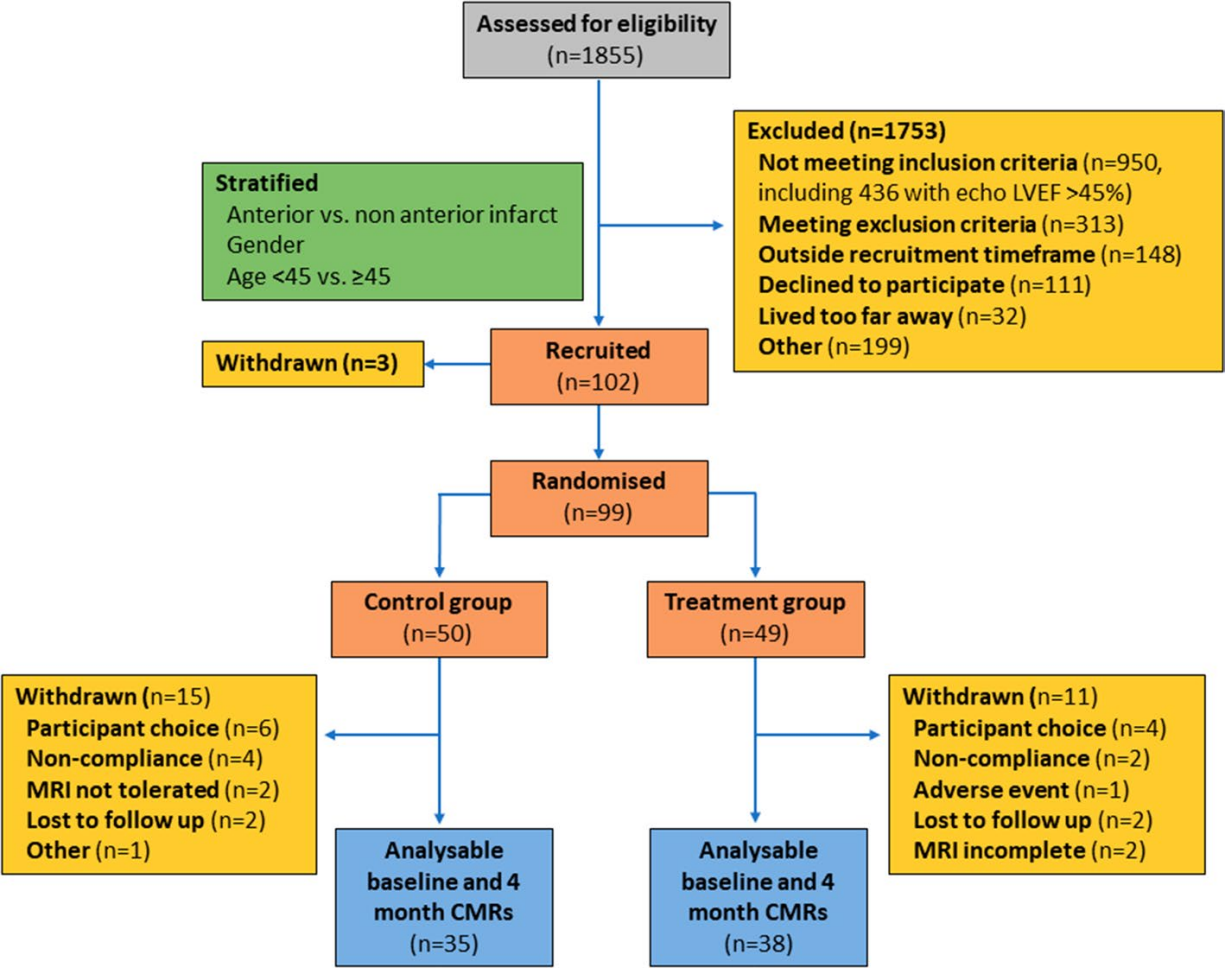
CONSORT (Consolidated Standards of Reporting Trials) diagram illustrating recruitment and patient flows in the DREAM trial. *LVEF* left ventricular ejection fraction

Following their index presentation with STEMI, participants underwent CMR imaging at 5–7 days (baseline) and at 4 months (follow up, Fig. 1). CMR imaging was carried out on a 1.5-Tesla scanner at each of the four participating centres with retrospective electrocardiographic gating and dedicated cardiac receiver coils. Following standard pilot/localiser images, functional cine images were acquired according to standard clinical protocols using a steady-state free precession (SSFP) pulse sequence, in the three long-axis views and in contiguous short-axis slices covering the left ventricle. Late gadolinium enhancement (LGE) imaging was performed in the same slice positions using a segmented inversion-recovery gradient echo sequence.

CMR analysis was undertaken blinded to all clinical details, randomisation and temporal order of scans, and data presented according to the 17-segment American Heart Association segmentation model [2]. For functional assessment, endocardial and epicardial borders were manually contoured on contiguous short-axis LV slices, excluding papillary muscles and trabeculae at end-diastole and at end-systole, using CVI^42®^ (Circle Cardiovascular Imaging Inc. Calgary, Canada) [51]. For assessment of strain, SSFP cine images were analysed using feature tracking software (Medis Qstrain 2.0, Medis Medical Imaging Systems, Leiden, The Netherlands). Epicardial and endocardial borders were manually contoured in the three long-axis views and three selected short-axis views (basal, mid and apical). By automated propagation of contours through the cardiac cycle, peak circumferential and longitudinal strain were derived, globally and at the segmental level. The software automatically generated strain in endocardial, mid-myocardial and epicardial layers, which were averaged for transmural strain. Intraobserver and interobserver agreement for strain analysis in our centre are excellent as previously reported [28]. For LGE analysis, areas of hyperenhancement were quantified using the full-width half maximum method, expressed in grams and as percentage of LV mass (CVI^42®^_,_ Circle Cardiovascular Imaging Inc. Calgary, Canada) [11]. Additionally, infarct transmurality was graded visually on a 4-point scale as described previously (0–no LGE, 1–1–25%, 2–26–50%, 3–51–75%, 4–75–100%), with an LGE score being calculated as the sum of scores from all segments divided by 17 (yielding a maximum score of 4) [29]. Segments with LGE on the baseline scan were classified as infarct-related, and those without were classified as remote.

### Statistical analysis

The original sample size was calculated on the basis of an improvement in LVEF with CRIC. For the present analysis, a sample size of 35 patients per group was needed to afford 90% power with α level 5% to detect a 25% difference in global longitudinal strain at follow up. Normality was assessed using histograms and Q–Q plots. Continuous data are expressed as mean (± standard deviation) or median (interquartile range) and compared with Student *t* tests or Mann–Whitney tests as appropriate. Binary data are expressed as numbers (percentages), and comparisons were performed with the Chi-square or Fisher exact test. For comparison of strain at the patient level, strain values for each patient were averaged in infarcted and remote segments respectively and compared with Mann–Whitney or Student *t* test as appropriate. To investigate whether the group difference in infarcted and remote segments varied between layers, we used individual segment strain values and a fitted linear mixed model which accounted for the dependency of segments from the same patients. We modelled ‘change from baseline’ with adjustment for baseline strains. Separate models were developed for longitudinal and circumferential strain. Given the exploratory nature of this post-hoc analysis, we did not adjust for multiple testing. In addition, we calculated AUC (area under the ROC curve) to examine the efficacy of strain parameters to predict adverse remodelling at follow up (defined as end-diastolic volume increase ≥ 20% and/or end-systolic volume increase ≥ 15% with ejection fraction ≤ 40%) [3, 4]. All statistical analyses were conducted using Medcalc 9.5.2.0 and SAS 9.4. Statistical tests were 2-tailed, and *p* < 0.05 was considered significant.

## Results

### Study participants

As previously described, patients in the two groups (control *n* = 35, treatment *n* = 38) were well matched with regards to baseline demographic and clinical characteristics (Table 1) [9]. The majority of infarcts involved the left anterior descending coronary artery. All participants received drug-eluting stents. Discharge medication was similar between the two groups, with high uptake of optimal medical therapy. The median time between hospital admission and baseline CMR assessment was 4.6 days (3.2–5.9) in the control group and 4.5 days (3.3–4.9) in the treatment group (*p* = 0.41). Follow up imaging was performed at a median of 122 days (120–126) after admission in the control group and at 123 days (122–125) in the treatment group (*p* = 0.50). All CMR images were of sufficient quality and no images were excluded from analysis (Fig. 1).

**Table 1:** Demographic, clinical and imaging parameters

	Control	Treatment	*p* value
	(*n* = 35)	(*n* = 38)	
Demographic			
Age (years)	58.9 (11.9)	58.7 (9.9)	0.92
Sex (% male)	29/35 (82.9%)	31/38 (81.6%)	1
BMI	27.6 (4.0)	27.7 (4.5)	0.88
BSA (m^2^)	2.0 (0.2)	1.9 (0.2)	0.1
Clinical characteristics			
Anterior STEMI	28/35 (80.0%)	27/38 (71.1%)	0.43
Multiple vessel disease	5/33 (15.2%)	7/37 (18.9%)	0.76
Baseline systolic blood pressure (mmHg)	122.7 (20.9)	120.2 (19.3)	0.59
Baseline diastolic blood pressure (mmHg)	76.2 (15.9)	73.7 (14.1)	0.49
Baseline heart rate (bpm)	75.8 (18.4)	76.5 (14.7)	0.85
Follow up systolic blood pressure (mmHg)	124.0 (21.2)	122.4 (17.4)	0.73
Follow up diastolic blood pressure (mmHg)	75.4 (14.8)	75.1 (7.3)	0.91
Follow up heart rate (bpm)	62.3 (7.6)	61.4 (15.3)	0.75
Baseline CMR parameters			
LVEF (%)	43.7 (6.7)	43.2 (7.2)	0.75
LVEDV (ml)	182.0 (139.4–193.8)	189.5 (158.8–218.4)	0.13
LVESV (ml)	101.7 (87.0–108.9)	107.9 (82.4–135.9)	0.17
Myocardial mass (g)	130.0 (110.0–147.8)	130.0 (109.6–154.3)	0.88
Presence of MVO	31 (88.6%)	27 (71.1%)	0.09
Infarct size (% of LV mass)	21.9 (16.9–35.4)	21.4 (13.0–32.3)	0.53
Global longitudinal strain	− 16.5 (2.3)	− 16.8 (3.2)	0.71
Global circumferential strain	− 16.9 (3.4)	− 16.8 (3.5)	0.91
Follow up CMR parameters LVEF			
(%)	48.3 (6.7)	48.4 (8.4)	0.94
EDV (ml)	183.5 (36.3)	197.0 (52.2)	0.21
ESV (ml)	96.1 (26.5)	104.5 (41.0)	0.3
Myocardial mass (g)	108.2 (98.9–127.4)	107.0 (90.2–124.9)	0.76
Infarct size (% of LV mass)	17.6 (13.7–22.6)	15.2 (8.5–23.3)	0.59
Global longitudinal strain	− 18.4 (3.4)	− 19.2 (4.0)	0.38
Global circumferential strain	− 19.8 (3.9)	− 19.0 (4.1)	0.37

Results are shown as mean (SD) or median (Q1–Q3) for continuous variables and as number of patients (percentage) for categorical variables

*LVEDV* left ventricular end-diastolic volume, *LVEF* left ventricular ejection fraction, *LVESV* left ventricular end systolic volume, *LVM* left ventricular mass, *MVO* microvascular obstruction, *STEMI* St-elevation myocardial infarction

### Global CMR parameters

At baseline, volumetric parameters were comparable in both groups (Table 1): there was no difference in LV ejection fraction at baseline or at follow up. Similarly, there were no differences in infarct size at either timepoint (at baseline, 33.5 ± 18.8 g in controls versus 32.6 ± 25.3 g in the treatment group, *p* = 0.86, and at follow up, 19.9 g (15.0–23.1) versus 15.2 g (7.8–29.9), respectively, *p* = 0.37). At baseline, 43.0 ± 15.2% of segments showed evidence of LGE in the control group and 41.0 ± 19.3% in the treatment group (*p* = 0.47), and at follow up, 38.7 ± 13.0% and 34.2 ± 19.3%, respectively (*p* = 0.10). At both timepoints, LGE score was comparable in the two groups (at baseline 1.12 ± 0.53 versus 1.03 ± 0.39, respectively, *p* = 0.52, and at follow up, 0.84 ± 0.39 versus 0.71 ± 0.27, respectively, *p* = 0.25). Microvascular obstruction was more prevalent in the sham group but this did not reach statistical significance.

**Fig. 2:**
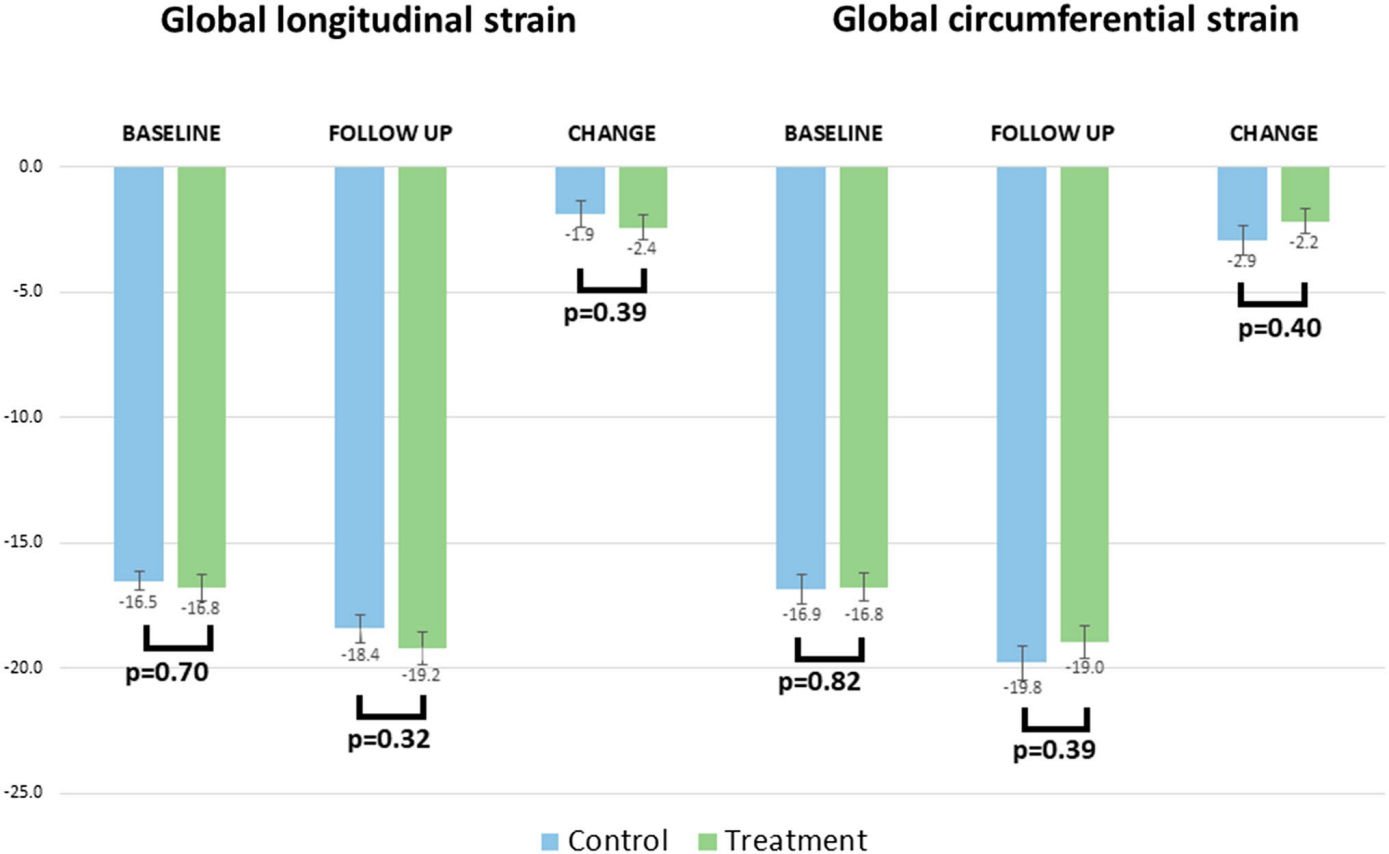
Global longitudinal and circumferential strain for control (blue) and treatment (green) groups at baseline and follow up, with absolute change between timepoints. Error bars showing mean standard error

Analysis of myocardial strain revealed no between-group differences at baseline in either global longitudinal strain or global circumferential strain (Fig. 2). Similarly, at follow up, there were no differences between control and treatment groups (change in global longitudinal strain −1.9±3.1 versus −2.4±3.0, respectively, *p*=0.39; change in global circumferential strain −2.9±3.4 versus −2.2±3.1, *p*=0.40).

### Patient-level analysis myocardial strain in infarcted versus remote segments

**Fig. 3:**
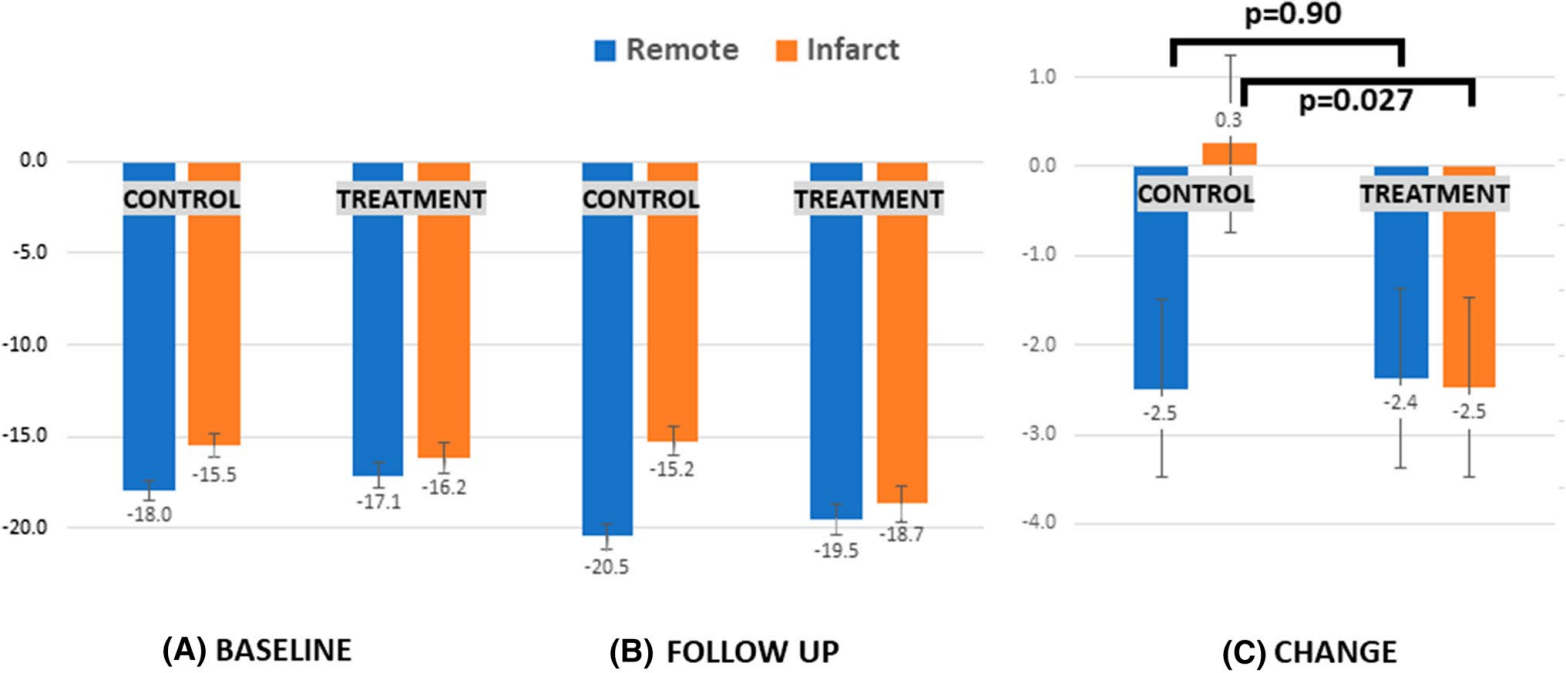
Longitudinal strain (transmurally) for control (*n* = 35) and treatment (*n* = 38) groups at **A **baseline (leftmost panel) and **B** follow up (middle panel), with **C** absolute change between timepoints (rightmost panel). Blue bars denote remote regions and orange bars denote infarcted regions. Error bars showing mean standard error

Further patient-level analysis was carried out, evaluating infarcted (*n* = 73) and remote (*n* = 73) territories. At baseline, longitudinal strain was found to be lower in infarcted regions than in remote, with no between-group differences (Fig. 3A). At follow up, in the remote territories, there was a significant improvement in longitudinal strain relative to baseline in both arms (Fig. 3B, C). By contrast, in the infarct-related regions of the control arm, there was no improvement in longitudinal strain (− 15.5 ± 4.0 at baseline to − 15.2 ± 4.7 at follow up, *p* = 0.81). However, in the infarct-related regions of the treatment arm, there was a significant improvement in longitudinal strain (from − 16.2 ± 5.2 at baseline to − 18.7 ± 6.3 at follow up, *p* = 0.0006). Consequently, at follow up in the treatment arm, there was no significant disparity between infarcted and remote regions (− 18.7 ± 6.3 versus − 19.5 ± 5.2, respectively, *p* = 0.46), i.e. there was near normalisation of strain within infarcted territories. However, in the control arm, consistent with a lack of improvement in infarcted regions, there remained a significant disparity in longitudinal strain between these and remote regions at follow up (− 15.2 ± 4.7 versus − 20.5 ± 4.0, respectively, *p* < 0.0001). Examining the change in strain from baseline to follow up (Fig. 3C), there was a significant difference in the infarct-related regions of both groups (+ 0.3 ± 5.6 in the control group versus − 2.5 ± 3.6 in the treatment group, *p* = 0.027), with no difference in the remote regions of both groups (− 2.5 ± 3.5 versus − 2.4 ± 4.3, respectively, *p* = 0.90).

**Fig. 4:**
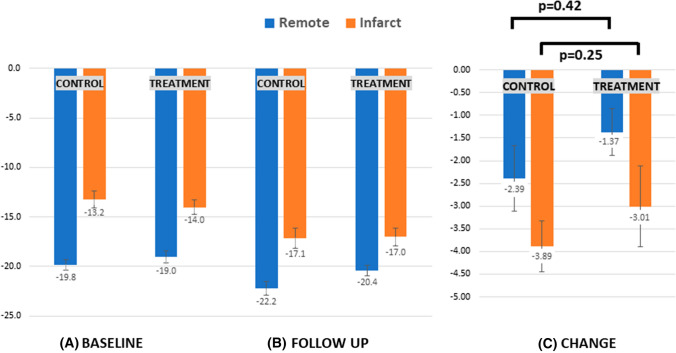
Circumferential strain (transmurally) for control (*n* = 35) and treatment (*n* = 38) at **A** baseline (leftmost panel) and **B** follow up (middle panel), with **C** absolute change between timepoints (rightmost panel). Blue bars denote remote regions and orange bars denote infarcted regions. Error bars showing mean standard error

Circumferential strain at baseline was significantly lower in infarcted regions than in remote, with no between-group differences (Fig. 4A). At follow up, circumferential strain improved significantly from baseline in infarcted and remote myocardium in both arms (Fig. 4B). Although the magnitude of change was greater in the control group, this did not reach statistical significance in patient-level analysis (Fig. 4C).

### Segment-level analysis of myocardial strain in myocardial layers

**Fig. 5:**
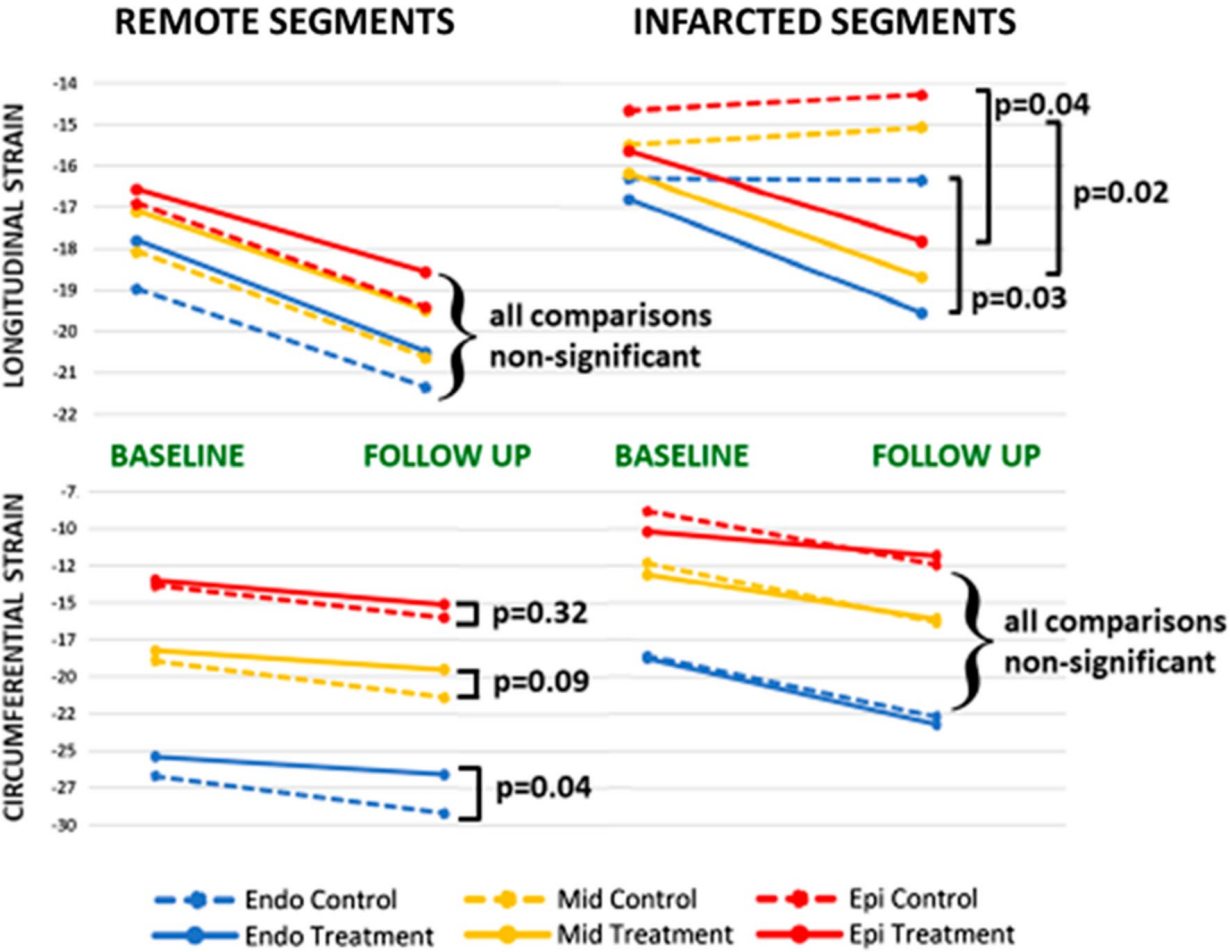
Mean longitudinal strain (upper two panels) and circumferential strain (lower two panels) at each timepoint depicted by myocardial layer  -red denoting epicardial, yellow, mid-myocardial and blue, endocardial. Solid lines denote the treatment group and dashed lines, the control group. *P* values derived from mixed effects model accounting for differences in strain at baseline. *Endo* endocardial, *Epi* epicardial, *Mid* mid-myocardial

A total of 1168 segments (475 infarcted and 693 remote) were available for segmental analysis with the mixed effects model. In remote segments, there was improvement in longitudinal strain in all three myocardial layers, with no between-group differences (Fig. 5, top left panel). By contrast, in segments with infarction, consistent with patient-level analysis, there were significant between-group differences in longitudinal strain, apparent in all three myocardial levels (Fig. 5, top right panel). Specifically, whereas longitudinal strain improved in all three layers of the CRIC arm, in the control arm, there was no significant change in any layer.

When circumferential strain was assessed, in contrast to longitudinal strain, in the infarcted segments, there was significant improvement in all three layers of both groups, with no between-group differences (Fig. 5, bottom right panel). However, in remote segments, there was a significant between-group difference: although circumferential strain improved from baseline to follow up, there was a higher increment in the endocardial layer of the control group than the corresponding layer in the treatment group (i.e. an attenuated increase in the latter, (− 1.2 ± 4.4 versus − 2.5 ± 4.0, p = 0.038, Fig. 5 bottom left panel). A trend towards higher increment in the mid-myocardial layer of the control group was also apparent (*p* = 0.09) with no difference in the epicardial layer (*p* = 0.32).

### Strain parameters in relation to remodelling

Eight patients (11%) developed adverse remodelling at follow up (3 in the control group and 5 in the treatment group, *p* = 0.53). The ability of baseline infarct size to predict adverse remodelling at follow up did not reach statistical significance (Table 2). However, although baseline GLS was not of predictive value, baseline GCS was predictive of adverse remodelling (threshold <  − 15.7, 88% sensitivity, 67% specificity, AUC 0.82 ± 0.06, *p* < 0.0001; *p* = 0.015 for difference with GLS). When examined separately in infarcted versus remote territories, circumferential strain in both remained predictive of adverse remodelling. By contrast, with longitudinal strain, diminished strain in infarcted regions predicted subsequent adverse remodelling but strain in remote territories was not of predictive value.

**Table 2:** Receiver operative characteristic (ROC) curve analysis of infarct size and strain parameters in relation to adverse remodelling

	AUC	Standard error	95% CI	*p* value
Baseline infarct size	0.71	0.13	0.46–0.95	0.1
GCS	0.82	0.06	0.70–0.95	< 0.0001
Circumferential strain-remote	0.73	0.09	0.62–0.83	0.01
Circumferential strain-infarct	0.75	0.08	0.60–0.90	0
GLS	0.63	0.1	0.51–0.74	0.19
Longitudinal strain-remote	0.52	0.14	0.23–0.80	0.92
Longitudinal strain-infarct	0.68	0.08	0.53–0.83	0.02
Follow up				
Infarct size	0.7	0.09	0.58–0.80	0.04
GCS	0.96	0.02	0.91–1.00	< 0.0001
Circumferential strain-Remote	0.87	0.06	0.74–0.99	< 0.0001
Circumferential strain-Infarct	0.89	0.04	0.80–0.98	< 0.0001
GLS	0.85	0.06	0.73–0.98	< 0.0001
Longitudinal strain-Remote	0.76	0.09	0.58–0.94	0.01
Longitudinal strain-Infarct	0.8	0.06	0.67–0.92	< 0.0001

*AUC* area-under-curve, *GCS* global circumferential strain, *GLS* global longitudinal strain

On evaluation of strain parameters on the follow up scan in relation to remodelling, all parameters (longitudinal and circumferential in remote and infarcted territories) were associated with adverse remodelling (Table 2). Circumferential strain remained most closely correlated with adverse remodelling (AUC 0.96 ± 0.02, *p* < 0.0001, with strain values <  − 16.0 having 100% sensitivity and 91% specificity for identifying patients with adverse remodelling).

## Discussion

This post-hoc analysis of the DREAM trial reveals that although CRIC does not alter infarct size or global LV indices (volumetry/strain), it is associated with altered regional strain in infarct-related and remote territories. The use of CRIC was associated with improvement in longitudinal strain in infarcted territories and an attenuated increase of circumferential strain in both infarcted and remote territories.

It remains unclear what underlies the disappointing failure to translate promising pre-clinical/early-phase clinical findings into hard clinical endpoints: potential reasons are extensively discussed in the literature.[20, 21, 23, 30] Interspecies differences mean that animal models may not fully replicate infarction in humans: whereas animal models utilise young, healthy animals, human disease is characterised by chronic atherosclerosis and the influence of risk factors such as diabetes, hypertension and hyperlipidaemia. Outcomes in clinical trials may be confounded by the influence of medications which can interfere with cardioprotection (e.g. P2Y purinoceptor 12 inhibitors or glyceryl trinitrate) or influence healing and remodelling independent of infarct size reduction (e.g. angiotensin-converting enzyme inhibitors and angiotensin II-receptor blockers).[17] Furthermore, improvements in reperfusion therapy (producing better and faster recanalisation) to attenuate infarct size and adjuvant pharmacotherapies to limit adverse remodelling may be so effective that no additional intervention with RIC may impact clinical outcome. Pre-infarct angina may also afford cardioprotection through the development of coronary collaterals or through a preconditioning-like effect. To date, most clinical studies have been small-scale and statistically underpowered, using surrogate measures rather than hard clinical endpoints. Even in the CONDI-2/ERIC-PPCI trial (*n*=5401), the utility of RIC may also have been limited by favourable patient factors (short symptom-to-PPCI time [median 3 hour] and spontaneous recanalisation at admission [TIMI 2-3 flow] in approximately 20% of participants). Greater benefit may be seen with RIC in higher risk patients, such as those with heart failure or large anterior infarcts [36].Another potential confounder is variation in the conditioned tissue mass: RIC administered to a leg may provide a greater stimulus than on a forearm: in a murine model, dual hindlimb RIC [with a greater mass of ischaemic/reperfused tissue] led to greater cardioprotection than single hindlimb RIC [33]. Similarly, a clinical trial utilising lower limb RIC demonstrated robust reduction in cardiac mortality and heart failure hospitalisation in contrast with many trials utilising forearm RIC which have failed to demonstrate clinical benefit [14]. However, another murine model found that one and two hindlimb preconditioning were equally protective, and a randomised clinical trial involving lower limb remote ischaemic per/postconditioning in 93 patients with anterior STEMI demonstrated no difference in myocardial salvage index or infarct size [26, 53].

In the CONDI-2/ERIC-PPCI trial, remote ischaemic perconditioning neither reduced infarct size (as assessed by 48-h troponin release or by CMR [*n* = 110]) nor improved the primary clinical endpoint (composite of cardiac mortality and heart failure hospitalisation at 12 months) [13, 18]. However, previous RIC studies have suggested the presence of discordant effects on infarct size and clinical outcomes. In the RIC-STEMI trial (*n* = 516), there was no reduction in infarct size with adjunctive RIC (based on 48 h troponin release) but the primary composite outcome of cardiac death and heart failure hospitalisation was significantly reduced (hazard ratio 0.35, 95% CI 0.15–0.78, median follow up 2.1 years) [14]. Other studies indicate the potential for additional cardioprotection by extending the period of conditioning beyond the time of ischaemia/reperfusion. A CMR study of postconditioning immediately after reperfusion in PPCI-treated STEMI patients (*n* = 122) showed no reduction in infarct size but at 1 year, adverse remodelling was reduced, especially in those with microvascular obstruction [50]. In the LIPSIA-CONDITIONING trial (*n* = 696), combined RIC and postconditioning resulted in greater myocardial salvage than conventional PPCI alone, and with extended follow up (median 3.6 years), a reduction in cardiac death, reinfarction and new congestive cardiac failure (10.2 versus 16.9%, *p* = 0.04) [8, 47]. However, in this trial, postconditioning alone did not reduce MACE (14.1 versus 16.9% in controls, *p*-0.41), and other postconditioning studies have also reported neutral outcomes [10, 16, 46]. Nonetheless, taken together, these results indicate that although infarct size may not be reduced with RIC, the subsequent remodelling process may be altered for therapeutic gain.

To our knowledge, ours is the only trial to evaluate CRIC post-STEMI: to date, no other clinical trial has evaluated late, repetitive RIC post-STEMI, though the CORIC-MI (*n* = 200) and i-RIC (*n* = 4700) trials will incorporate CRIC following STEMI (with additional per/postconditioning) [45, 63]. However, CRIC has been investigated in experimental animal studies. In an animal model of ischaemia/reperfusion injury, CRIC administered for 28 days resulted in improved LV remodelling and also survival (at 84 days), and, consistent with our findings, no change in infarct size [55]. Importantly, in our trial, CRIC was not commenced till day 3 post-MI. Given the critical 48 h timeframe ascribed for reperfusion injury when infarct size attenuation may be targeted therapeutically, any benefit from ‘late’ CRIC likely involves mechanisms distinct from infarct size reduction [34, 62]. Previous work has shown that cardioprotection may be mediated by dialysable humoral factors which can circulate for 6 days after a RIC stimulus [24]. Our data indicate that the benefits of CRIC are unlikely to involve haemodynamic parameters, which remained comparable in both groups at follow up (Table 1). Although microvascular obstruction, a known predictor of adverse remodelling, was more prevalent in the sham group, this did not reach statistical significance.

Nonetheless, if CRIC is beneficial, why was there no observed effect on LVEF or other volumetric indices of remodelling? Global volumetric indices integrate the function of infarcted and remote regions, and hence may be insensitive to regional dysfunction, particularly if compensatory mechanisms subsequently normalise global performance. Furthermore, gross volumetric change may only occur late in the remodelling process, in a maladaptive state beyond the point of no return. By contrast, strain imaging may prove more sensitive, identifying subtle, early and potentially reversible regional derangements in those at risk of at risk of adverse remodelling [37]. Impairment of longitudinal strain has been shown to occur early in many pathological disease states, preceding the onset of overt systolic dysfunction [7, 27].

Strain analysis may also provide pathophysiological insights into STEMI-induced remodelling. The myocardium comprises a complex spatial orientation of fibres, with subendocardial fibres orientated in a right-handed helix and subepicardial fibres, in a left-handed helix, with mid-myocardial fibres arranged circumferentially [15, 49]. This physiological arrangement is mechanically advantageous, providing energetic efficiency, with optimum redistribution of shear forces [52]. The subendocardial fibres are especially vulnerable to the effects of ischaemia, and post-MI, longitudinal function declines first [44, 54, 61]. This may be compensated by augmenting short-axis function, mediated by circumferential fibre shortening [54]. Clinical evidence suggests that whereas GLS is a better predictor of MACE (driven by ischaemic/scar-related events), GCS better predicts adverse remodelling [7, 25]. It is likely that circumferential function initially compensates for longitudinal dysfunction (and restrains ventricular dilatation), but subsequently may decline, with ensuing dilatation and adverse remodelling [48]. However, even prior to decompensation, augmented circumferential function may be maladaptive, increasing cardiac workload and shear stress in damaged regions. Myocardial stretching/thinning predisposes to the development of sphericity, which reduces mechanical advantage and is associated with adverse outcome [39, 41].

Our data show increased circumferential strain in remote as well as in infarcted segments. Studies using CMR diffusion tensor imaging have shown reorientation of fibres in remote myocardium post-MI [35, 58, 59]. Animal models show that local strain patterns may guide the alignment of collagen fibres during scar formation [12]. Hence, in the chronic phase post-STEMI, altered regional mechanics may influence the propensity to adverse remodelling. Intuitively, rather than augmenting a compensatory, potentially maladaptive mechanism, correction of the initial pathological derangement may be preferable. Our data indicate that CRIC may target the initial derangement in longitudinal function, minimising the disparity in longitudinal strain between infarcted segments and adjacent viable myocardium and lessening the requirement for circumferential compensation. This may prove mechanically and energetically advantageous and potentially mitigate against the development of heart failure [38].

### Study limitations

This study has several limitations. As CRIC was self-administered in participants’ homes, it was not possible to objectively verify correct application of the device and achievement of a satisfactory postconditioning stimulus. The present work was a non-prespecified post-hoc analysis and a small number of participants were involved: hence, the results should be interpreted with caution. The study was not powered to determine potential improvements in adverse remodelling, as defined by global parameters. However, as a proof-of-concept study it serves the purpose of hypothesis generation. Whether the observed changes in regional strain translate into a reduction in adverse remodelling and altered clinical outcome warrants exploration in larger-scale prospective studies. Our data do not elucidate the mechanisms underlying observed changes in strain, albeit demonstrating that these do not involve infarct size reduction or changes in haemodynamic parameters.

## Conclusions

Our analysis suggests that the beneficial effects of RIC may involve mechanisms distinct from infarct size limitation. This warrants further investigation in prospective studies as well as in analyses of imaging datasets acquired from previously studied RIC cohorts. Further study is required to determine whether a multi-target approach combining CRIC with ischaemic perconditioning interventions may afford a more effective, synergistic cardioprotective strategy.

## Abbreviations

CMR: Cardiac Magnetic Resonance Imaging
CRIC: Chronic Remote Ischaemic Postconditioning
DREAM: Daily Remote Ischaemic Conditioning Following Acute Myocardial Infarction
GCS: Global Longitudinal Strain
GLS: Global Circumferential Strain
LVEF: Left Ventricular Ejection Fraction
MI: Myocardial Infarction
P-PCI: Primary Percutaneous Coronary Intervention
STEMI: ST-Elevation Myocardial Infarction
RIC: Remote Ischaemic Conditioning
STEMI: ST-Elevation Myocardial Infarction
